# Determining whether Community Health Workers are ‘Deployment Ready’ Using Standard Setting

**DOI:** 10.29024/aogh.2369

**Published:** 2018-11-05

**Authors:** Celia Taylor, Basimenye Nhlema, Emily Wroe, Moses Aron, Henry Makungwa, Elizabeth L. Dunbar

**Affiliations:** 1 Division of Health Sciences, University of Warwick, UK; 2 Partners In Health, Neno, MW; 3 Division of Global Health Equity, Department of Medicine, Brigham and Women’s Hospital, Boston, MA, US; 4 Department of Global Health and Social Medicine, Harvard Medical, Boston, MA, US

## Abstract

**Background:**

Community Health Workers (CHWs) provide basic health screening and advice to members of their own communities. Although CHWs are trained, no CHW programmes have used a formal method to identify the level of achievement on post-training assessments that distinguishes “safe” from “unsafe”.

**Objectives:**

The aim of this study was to use Ebel method of standard setting for a post-training written knowledge assessment for CHWs in Neno, Malawi.

**Methods:**

12 participants agreed the definitions of a “just-deployment ready” and an “ideal” CHW. Participants rated the importance and difficulty of each question on a three-point scale and also indicated the proportion of “just-deployment ready” CHWs expected to answer each of the nine question types correctly. Mean scores were used to determine the passing standard, which was reduced by one standard error of measurement (SEM) as this was the first time any passing standard had been employed.

The level of agreement across participants’ ratings of importance and difficulty was calculated using Krippendorf’s alpha. The assessment results from the first cohort of CHW trainees were analysed using classical test theory.

**Findings:**

There was poor agreement between participants on item ratings of both importance and difficulty (Krippendorf’s alphas of 0.064 and 0.074 respectively). The pass mark applied to the assessment, following adjustment using the SEM, was 53.3%. Based on this pass mark, 68% of 129 CHW trainees were ‘clear passes’, 11% ‘borderline passes’, 9% ‘borderline fails’ and 12% ‘clear fails’.

**Conclusions:**

Determining whether a CHW is deployment-ready is an important, but difficult exercise, as evidenced by a lack of agreement regarding question importance and difficulty. Future exercises should allow more time for training, discussion and modification of ratings. Based on the assessment, most CHWs trained could be considered deployment-ready, but following-up their performance in the field will be vital to validate the pass mark set.

## Introduction

Community Health Workers (CHWs) provide a wide range of services including basic health promotion, counselling and care within the communities in which they live, providing a critical link between communities and health systems [1,2]. CHWs are often seen as part of the solution to the shortage of health workers and lack of universal access to healthcare in low- and middle-income settings [3,4]. While there is accumulating evidence of the effectiveness of CHW programs, particularly for single disease areas such as screening for tuberculosis, adherence and retention for patients with HIV, or increasing access to maternal health services [5,6,7,8,9], CHW programmes are not always as effective as they could be [10,11]. There are a number of potential reasons for sub-optimal effectiveness, including insufficient or poor quality training [10,11], or because CHWs have difficulties in coping with the volume and complexity of the tasks asked of them [12]. Taken to the extreme, and in common with the risks associated with receiving care from any healthcare worker, it is plausible that CHWs cause unintended but preventable harm to their clients, particularly if they are not coherently included in the broader health system [10].

A CHW’s initial training should prepare them for their role in service delivery and ensure they have the skills required to provide safe, effective and quality care [13]. Post-training assessments of CHWs’ knowledge and skills are recommended to evaluate CHWs’ learning during such training [14]. However, to determine whether a CHW is ready to be deployed into the field (often to work by themselves), a program provider needs to know what minimum score is indicative of ‘minimum competence’ or readiness for safe deployment. The need for such a minimum score, or pass mark, can be implied from the studies of Workman et al. [15] and Kalyango et al. [16] Workman et al. [1]report that CHWs in Brazil achieved a mean score of 41% on an assessment of knowledge about childhood cancer, noting that “without adequate training in and knowledge of the warning signs and symptoms of childhood cancer, it is unlikely that … CHWs can … thereby begin to decrease the discrepancies in terms of survival rates for childhood cancer” (p. 183–4). However we do not know what score would have been required to reverse this conclusion. Kalyango et al. [16], meanwhile, report a similar average knowledge score for pneumonia (median 40%) for CHWs in Uganda, but do not conclude that this is an insufficient level of knowledge for deployment. Anecdotal evidence suggests that post-training assessments appear fairly common, but we were only able to find three studies in which a pre-determined pass mark for a post-training assessment of knowledge was reported [17,18,19]. In two of these studies [17,18,19] it is not clear how the pass mark was determined, while in the third [20] the authors report that their 50% cut-off to determine competency for their written assessment of maternal, neonatal and child health was arbitrary and not “scientifically driven”. Furthermore, Abrahams-Gessel reports that the initial pass mark of 70% for the knowledge test on cardiovascular risk had to be reduced to 60% because of a very low pass rate amongst the first cohort of CHWs to take the assessment, and that there was no agreement between supervisors on “the level of competency that was expected to be achieved” (Abrahams-Gessel S., [18] p. 133). A 70% pass mark was also used in the study of Accredited Social Health Activists (ASHAs) in India with the assessment using true-false style questions, with over 20% of ASHAs failing to meet this standard [19]. The authors of this study also emphasise the potential risk to client safety if “valuable information is provided in error or omitted [during] counselling” because “many ASHAs lack the essential knowledge to perform their jobs well [19]” (p. 27–8).

In other healthcare professions such as medicine, attempts are made to establish appropriate pass marks for assessments using a process known as standard setting [21,22,23]. There is no ‘gold standard’ method of standard setting, although the use of ‘absolute’ standards over fixed or relative standards is generally advocated by educationalists [24]. A fixed standard would be a pre-determined pass mark of 50%, for example, without consideration of the difficulty of the assessment or whether this score is a good reflection of the standard required for safe practice. With a relative standard, a pre-determined percentage of candidates pass each sitting of the assessment, without consideration of differences in the ability level of different cohorts or whether those that pass are actually safe (and vice versa). With an absolute standard, a group of knowledgeable individuals determine, for each sitting of the assessment, the minimum score that reflects safe practice, with all candidates who achieve this score passing the assessment. Absolute standards also help to ensure fairness across different sittings of an assessment with different content: a particularly hard exam paper would have a relatively low pass mark and vice versa. Two common methods of setting absolute standards for written assessments, Angoff [25] and Ebel [26], are described in **Box 1**.

**Box 1: Common methods of standard setting**

Each method requires a panel of appropriately qualified individuals (for example, faculty involved with the design and delivery of teaching) to make decisions on individual test items or the test as a whole. Initial decisions are usually be made independently and the results then averaged across panel members or followed by discussion amongst panel members to agree relevant standards (often known as a ‘Modified’ approach). Research suggests that at least six panel members would be required to achieve sufficient reliability with discussion and ten without [27]. For the Angoff and Ebel methods, the panel must begin by agreeing on the definition of a ‘minimally competent’ candidate on the assessment.

Angoff [25]: The panel provides the proportion of minimally competent candidates who would answer each item correctly. The mean standard across all items in the assessment provides the pass mark.

Ebel [26]: The panel rate each item in two dimensions: (1) importance (e.g. essential, important, useful to know) and (2) difficulty (e.g. easy, moderate, challenging). This process creates a number of different categories of question (with the examples given there would be nine categories). They then agree what proportion of minimally competent candidates would answer each category of item correctly and the relevant proportion applied to each item to provide its standard. The mean standard across all items in the assessment provides the pass mark.

As CHW programs become more professionalized and CHWs take on more roles and responsibilities (with ensuing increased potential risks for their clients), it would seem appropriate to extend best-practice methods of standard setting used elsewhere to CHWs. Such an approach would help to maximise the health gains that can be achieved by CHWs. However, to our knowledge, no assessments used to evaluate CHWs’ knowledge, skills or attitudes have been standard-set using established methods to date.

## Methods

### Aims

The aims of this paper are:

1. To describe the application of the Ebel standard setting process used for a post-training written assessment of knowledge within a CHW programme,
2. To provide a short-term evaluation of the standard setting process, and
3. To review CHWs’ performance on the first sitting of the standard set assessment and consider the implications of applying the pass mark set.

### Study setting

The setting for this study is the rural district of Neno, which is in the southwest zone of Malawi on the border with Mozambique. Partners In Health (PIH), a US-based non-governmental organization, has worked with the Ministry of Health in Neno for the last ten years to strengthen primary and secondary healthcare services, with a strong focus on the community. Their initial work included a ‘disease-specific’ CHW program, in which CHWs were assigned to individuals who had specific conditions – primarily HIV and tuberculosis. However, they have recently developed a new, integrated ‘household model’ or polyvalent approach to the CHW program. In the new model, CHWs will undertake the following tasks:

1. Timely case finding through education and screening for common, treatable conditions.
2. Linkage to care for symptomatic clients along with those identified through routine screening.
3. Ongoing support and accompaniment of patients in care, including adherence support, psychosocial support, and tracking of missed patient visits.
4. Health education for common health conditions and prevention and management of these conditions to optimize prevention, health services uptake, and health management behaviors in the household.

The new model is being rolled-out across Neno over a two-year period. Once rollout is complete, there will be around 1,100 CHWs; the majority of these CHWs were employed in the disease-specific program, but there will also be some new recruits. CHWs will attend monthly group meetings led by Senior CHWs (who in turn are supported and managed by Site Supervisors) and will also receive quarterly one-on-one mentorship and supervision visits from their Senior CHW.

**Figure 1:**
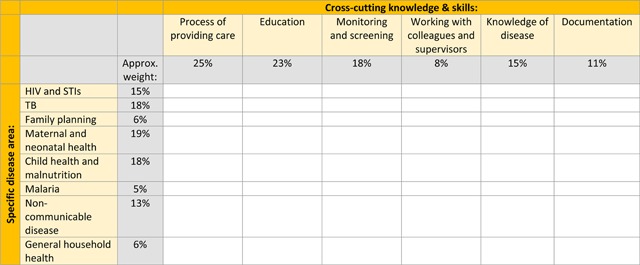
Assessment blueprint.

As part of implementation of the new model, new and existing CHWs will attend a five day initial training course, which will be followed by shorter refresher training sessions. The purpose of the initial training is to orient CHWs to the following: the new programme and its objectives (specific disease areas), their cross-cutting roles, their specific tasks (as listed above), and data collection and documentation requirements. This will be achieved by developing the knowledge, skills, and attitudes CHWs need to successfully perform the tasks listed above. The training is structured around the key point of interaction between CHWs and their clients: monthly home visits. Following the initial training, CHWs will complete a written knowledge assessment, which has been developed based on a blueprint defined by CHWs’ cross-cutting roles and disease areas (Figure 1). The assessment consists of 20 items (questions), including ‘fill in the blank’ with 1 or 3 answers required and single-best answer (1 from 3 or 5) items (Appendix 1). A total of 30 marks are available, with CHWs given 60 minutes to complete the assessment. The length of the test was determined pragmatically in relation to the length of the training course as a whole. The assessment was originally written in English, at which point all items underwent a quality review process using group discussion amongst the PIH development and implementation team. The assessment was subsequently translated into Chichewa and back-translated to ensure accuracy to the original English version.

### Describing the standard setting process

Standard setting process and panel: The standard setting process was undertaken in February 2017 as part of an orientation programme for those involved in training delivery (i.e. a training the trainer event). It was held at the PIH central office in the district of Neno. The standard setting panel was comprised of 12 individuals, including Malawi Ministry of Health/PIH staff involved in training delivery and PIH staff involved in development and implementation of the household model. All panel members have experience working with CHWs and of the requirements of the household model. All participants were given training about standard setting, the Ebel method and how it would work in practice.

The standard setting process followed good practice guidance [23] and used the Ebel method [26]. The panel first discussed its expectations of an ‘ideal’ and a ‘minimally competent’ CHW, in terms of the knowledge required to be exceptional and just safe to deploy, respectively. Each panel member then rated each assessment item independently. Answers were provided to participants. Item importance was rated on a three-point scale: very important, important or nice to know. The difficulty of each item for an ideal CHW was also rated on a three-point scale: easy, moderate or challenging. For items which carried more than one mark, difficulty was rated for a CHW obtaining full marks. As a result, each panel member classified each item into one of nine possible categories (Table 1). Panel members then individually estimated the expected proportion of *minimally competent* (i.e. just deployment-ready) CHWs that *would* answer an item in each of the nine categories correctly in an assessment.

**Table 1:** Standards by item importance and difficulty (/10).

	Very important	Important	Nice-to-Know

**Easy**	8.0	6.5	4.6
**Moderate**	5.6	4.6	2.6
**Challenging**	3.5	2.8	1.2

#### Determining the pass mark for the assessment

The mean proportion of minimally competent CHWs who would answer each category of item correctly was calculated across all panel members. These means were applied to each panel member’s item importance/difficulty category rating to give an item-level standard for each item for each panel member. The mean standard for each item across all panel members was used as the standard for that item. The weighted mean standard across all items was used as the initial pass mark for the assessment (termed the ‘Ebel pass mark’), with each item’s standard weighted by the number of marks allocated to it.

The group discussed the possible consequences of a CHW failing the assessment. Although the assessment was intended to help ensure client safety (i.e. mitigate false positive test results), there was a concern that CHWs may fail because they are not used to assessments, struggle with written tests or may narrowly miss the pass mark but be safe to be deployed with additional supervision and support (i.e. might be false negatives). It was therefore decided to reduce the Ebel pass mark by one standard error of measurement (SEM) to obtain the pass mark that would be applied for this assessment in practice (termed the ‘pass mark applied’). The SEM of the assessment was calculated using Cronbach’s alpha as the measure of reliability, based on the first cohort of CHW trainees to undertake the assessment (further explanation is provided in **Box 2**).

**Box 2: Explanation of statistical concepts**

Cronbach’s alpha: Measures the internal consistency of an assessment, or the extent to which the individual items are related with each other (and can, therefore, be used as a measure of reliability). The coefficient considers the correlations between candidates’ scores on all possible pairs of items and can range from 0 to 1. A coefficient of 0 implies that there are no relationships between item scores, while a coefficient of 1 implies that scores on one item can perfectly predict scores on all other items. Longer assessments tend to have higher Cronbach’s alpha coefficients.

Krippendorff’s alpha: Measures the inter-rater reliability of raters’ judgements. The method can be used with any number of raters, ordinal data (e.g. the three point rating scales used in the Ebel process) and it can also handle missing data [28]. The possible range of values is 0 to 1; a coefficient of 0 reflects that any agreement between raters is due to chance alone, while a coefficient of 1 reflects perfect agreement across all raters on all items.

Pearson’s correlation coefficient: Measures the strength of the linear relationship between two independent, normally distributed variables. The possible range of values is –1 to +1; a coefficient of –1 reflects a perfect negative linear relationship, a coefficient of +1 a perfect positive linear relationship (for both, if plotted on a scatter diagram, all points would lie exactly on a straight line drawn through the points) and a coefficient of 0 no linear relationship (a random distribution of points on the scatter diagram).

Spearman Brown prophecy formula: Can be used to predict how changing the number of items in an assessment would change its reliability, based on the reliability achieved in the original assessment [29] and the general rule that reliability increases as test length increases.

Standard error of measurement (SEM): Estimates the precision with which an individual candidate’s score has been measured by the assessment. If the candidate were to take the same assessment 100 more times with no learning effects, we would expect 68 of their scores to be within ±1 SEM of their original score.

It was decided that CHWs scoring between the Ebel pass mark and the pass mark applied (i.e. borderline passes) would be required to meet with their supervisors more frequently for their first few months in practice. CHWs scoring below the pass mark applied would be asked to attend additional training and retake the assessment prior to being deployed.

### Short-term evaluation of the standard setting process

#### Level of agreement between panel members

The overall level of agreement between panel members for each dimension (importance and difficulty) was estimated using Krippendorff’s alpha [28] in Stata v14 (see **Box 2**).

#### Relationship between item-level standards and candidate performance

To evaluate item-level standards, the standard set for each item was compared to the mean score for the item across the first cohort of CHW trainees. Ideally, performance in only the sub-group of borderline CHWs would be considered, but the relatively small size of the cohort (and hence the low number of borderline CHWs) means that estimates of performance in this sub-group are likely to be imprecise. We would expect mean cohort performance on each item to exceed the standard set for that item (cf. being approximately equal to it for the sub-group of borderline CHWs), but not so high as to suggest the standard was too low. We therefore calculated the percentage of items where the mean score was between 10 and 30 percentage points higher than the standard (noting there is no empirical ‘gold standard’ for this range). We would also expect a positive correlation between standards and mean cohort scores across all items and we therefore calculated the Pearson correlation coefficient between item-level standards and mean cohort scores (see **Box 2**).

### Candidate performance and implications of using the pass mark

We summarised the scores of the first cohort of CHW candidates taking the assessment. To consider the implications of using the pass mark applied, the proportion of CHWs in each of four score categories was calculated: clear fail (score < pass mark applied – 1 SEM), borderline fail (pass mark applied – 1 SEM < score < pass mark applied), borderline pass (pass mark applied < score < Ebel pass mark) and clear pass (score > Ebel pass mark).

## Results

### Characteristics of the standard setting panel

The twelve-member standard setting panel consisted of nine members from PIH and three from the Malawian Ministry of Health (25%). One-third of panelists were female, and the mean age was 37 years. The median years’ experience working in health in Neno District was 5.5, with a range of 1 year to 30 years. Five members were from the community team, three from the clinical team, and two from each of the environmental health and monitoring & evaluation teams. Regarding education, six had a diploma, four had a Bachelors degree, and two had a Masters degree.

### Describing the standard setting process

#### Definitions of a “minimally competent” and an “ideal” CHW

The standard setting panel discussed the expectations of minimally competent (just deployment-ready) and ideal CHWs in broad terms, rather than using specific applications of knowledge. Panel members found this a challenging task, and often cited skills and attributes that, whilst important, would be very difficult to assess in a written test. The broad expectations in relation to knowledge, used to define a minimally competent and an ideal CHW, are shown in **Box 3**.

**Box 3: Broad definitions of a minimally competent and an ideal CHW**

**Table:** 

Minimally competent	Ideal
Has to refer back to their notes	Consistent knowledge base
Only confident in knowledge of one disease area	Breadth of knowledge
Cannot transfer knowledge to a new situation/	Adapts to change and new situations
Repeats word-for-word from notes	
Makes preventable errors in documentation	Thorough
May not ask for help if unsure	Knows when and how to get help
	Learns quickly

#### Determining the pass mark for the assessment

Across all of the 20 items included in the assessment and all 12 panel members, 4.2% of item importance ratings were in the nice-to-know category, 27.7% were in the important category and 68.1% were in the very important category. These ratings suggest that the content of the items is appropriate for the CHW programme in Neno. Similarly, 59.9% of item difficulty ratings were in the easy category, 24.9% in the moderate category and 15.2% in the challenging category. These ratings suggest that the items are at the right level for an assessment of deployment-readiness (rather than an assessment used to rank CHWs, which would require a higher proportion of challenging items). Both sets of ratings can be found for each standard setter in the supplementary data file.

Each panel member provided their own estimation of the number out of ten just deployment-ready CHWs who would answer each of the nine categories of item correctly in an assessment. The mean of these estimates, shown in Table 1, was used to convert each panel member’s ratings of importance and difficulty into a numerical standard for each item. On average, the panel expected that only 12/100 minimally competent CHWs would answer a challenging, nice-to-know item correctly, but that 80/100 would do so for an easy, very important item.

Individual item standards, prior to weighting, ranged from 0.53 or 53% to 0.75 or 75% (standards for each item are shown in Appendix 1). Question #2 on the test ‘If a person tests positive for HIV, when should s/he start ARVs?’ was most frequently rated as easy and as very important and therefore had the highest standard (per mark available, prior to adjustment with the SEM) at 0.75. No items were rated as only nice-to-know by more than three panel members. Question #10 on the test ‘What should a CHW do following detection of a missed period?’ (with three answer options) was rated as challenging by six of the 12 panel members and therefore had a particularly low standard of 0.53.

The Ebel pass mark for the assessment, prior to adjustment using the SEM, was calculated as 61.6%. The SEM from the assessment undertaken by 129 CHWs in the first cohort was 8.1%, based on a Cronbach alpha coefficient of 0.78 and a standard deviation of CHWs’ scores of 17.5%. Subtracting the SEM from the Ebel pass mark gave 53.4%, or 16.02/30 marks. It was decided to round this down so that the pass mark applied was 16/30 marks (53.3%).

### Short-term evaluation of the standard setting process

#### Level of agreement between panel members

The level of agreement between panel members for ratings of both item importance and difficulty was barely better than chance, with Krippendorff’s alpha coefficients of 0.064 and 0.074 respectively. Had more time been available, the panel would have discussed their ratings and been given the opportunity to modify them, as in a ‘Modified Ebel’ standard setting process, which may have reduced the variability amongst panel members.

**Figure 2:**
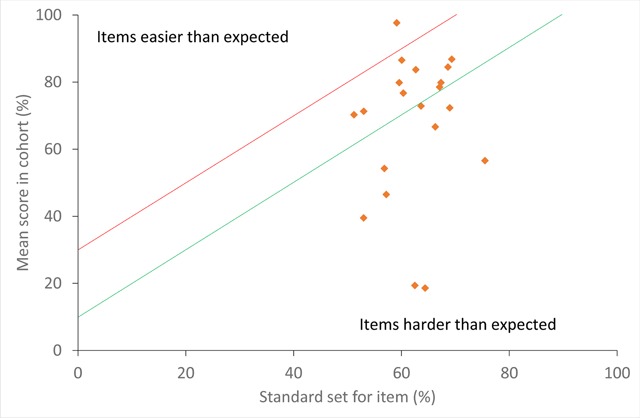
Relationship between standards set and item performance; Legend: The green diagonal line shows where the mean cohort score is 10 percentage points higher than the standard set and the red diagonal line 30 percentage points.

Relationship between item-level standards and candidate performance: The mean score of the cohort on an item was between 10–30 percentage points higher than the standard set for the item for 10/20 items (50%). The correlation between standards and mean cohort scores was poor (Pearson’s r = 0.13, p = 0.585), as shown in Figure 2. One item was much easier than predicted by standard setters (top of Figure 2), which asked about the correct use of ready-to-use therapeutic food to treat malnourished children (Question #15). Two items were particularly challenging compared to the standards set for them (bottom right corner of Figure 2); these asked about CHWs’ role in the mother and baby visit three days after birth (Question #11) and the main risk of a home birth (Question #19).

### Candidate characteristics

Of the 129 CHWs taking the assessment, 99 (77%) were female. Age and education data were available for 122 CHWs. Of these, the mean age was 39 years (range 23 to 67 years). 54 (44%) had not completed primary school, 28 (23%) had only completed primary school, 30 (25%) had completed junior secondary school (26 obtaining the Junior Certificate of Education) and the remaining 10 (8%) had completed senior secondary school and obtained the Malawi School Certificate of Education.

### Candidate performance and implications of using the pass mark

**Figure 3:**
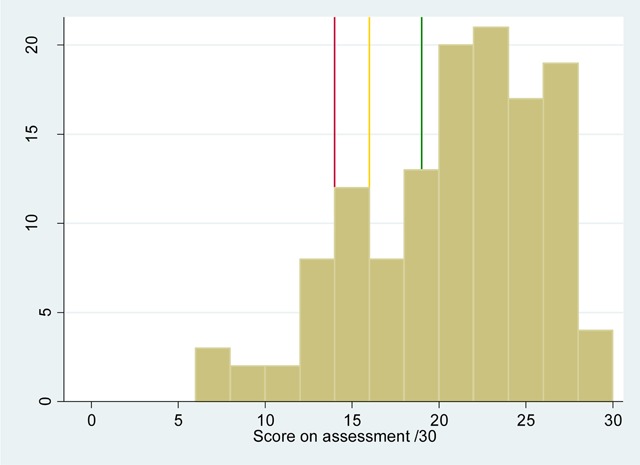
Histogram of CHWs’ scores; Legend: Red line (14/30) – boundary between clear fail and borderline fail; Yellow line (16/30) – boundary between borderline fail and borderline pass; Green line (19/30) – boundary between borderline pass and clear pass. The lines are not equally spaced as they have been rounded to the nearest whole number.

CHWs’ scores were calculated following a post-assessment item review process, in which the items with a low overall facility were checked to ensure papers had been correctly marked. Figure 3 shows a histogram of CHWs’ scores on the assessment. Twelve percent of the 129 CHWs were in the ‘clear fail’ category, scoring less than 14 marks/30 and a further 9% were in the ‘borderline fail’ category, scoring 14 or 15 marks/30. 11% were ‘borderline passes’, scoring between 16 and 18 marks/30 and 68% were ‘clear passes’, scoring at least 19 marks/30. Hence the overall pass rate was 79%, with 20% of CHWs considered ‘borderline’, i.e. about whom there is some uncertainty as to whether they should have passed or failed.

## Discussion

### Summary

Although we have not reported a novel approach to standard setting (the Ebel method itself was first reported in 1954) [26], to our knowledge this is the first reported attempt to describe a formal standard setting process for a post-training assessment of CHWs’ readiness for deployment (aim 1). Although the standard setting panel was of a reasonable size [27], there was very low agreement between panel members as to the importance and difficulty of the items included in the assessment (aim 2). As such, the passing standard itself was not very reliable. As also reported by Abrahams-Gessel [18], there seems to be a lack of agreement amongst trainers and program managers as to the expected level of competency. For nine of the 20 items, the mean cohort performance was below the standard set, suggesting that these items were harder in practice than rated by the panel members (aim 2). The assessment itself had good internal consistency (Cronbach’s alpha 0.78) given its relatively short length. A reliability of at least 0.8 is advocated as a minimum for ‘high stakes’ assessments [30], and the Spearman Brown prophecy formula (see **Box 2**) suggests that this could be achieved with around three additional items. One in five CHWs were in the borderline performance category (aim 3).

### Practical implications

Additional training, supervision and/or performance monitoring will be provided for the borderline pass CHWs (a policy that will be continued as roll out of the household model occurs in other districts). All CHWs participate in quarterly refresher trainings and the topic areas for such training could be identified by using the aggregate results from the assessment. For example, the two most challenging items in the assessment related to peri- and post-natal health. We might expect those CHWs newly recruited for the household model to have scored below their established peers in the post-training assessment (data were not available to test this hypothesis). If so, then a longer training period (currently five days) might be required for new recruits, although these CHWs may ‘get up to speed’ very quickly once in post. The standards and training curriculum for the items with large discrepancies between standards and performance should also be revisited if a similar pattern is seen across all CHW cohorts.

### Limitations

We used the terms ‘minimally competent’ and ‘just-deployment ready’ as the threshold to distinguish pass from fail. In a previous study, Ariff et al. [20] defined competency as “having sufficient knowledge and skills to comply with predefined clinical standards”. We asked panel members to construct their own definition although they found this challenging. It may also be the case that merely being ‘safe’ may not be sufficient to justify the costs of CHW programs, because CHWs are usually deployed in resource-constrained settings.

Ideally, more training for panel members would be provided and the process repeated. Such additional training could include facilitated discussion of a large number of practice items to help panel members reach a consensus on item difficulty and importance. The provision of example performance data so that the panel members had some prior knowledge of the type of responses that trainee CHWs might give to the items used would be very helpful for such training (as well as for the actual standard setting). In addition, including a facilitated discussion of all ‘live’ items would have been helpful to reduce the variability amongst panel members [27].

### Future work

We hope that this work can support others who wish to apply a similarly formal approach to setting pass marks for post-training assessments. Similar approaches can also be used for practical or clinical assessments, including for competencies such as communication skills. This is particularly important as post-training assessments become more routinely recommended and utilized [2], and as policymakers discuss professionalizing cadres of CHWs.

Studying the on-the-job performance of borderline pass CHWs (and contrasting it with that of the highest-scoring CHWs) would also provide a better indication of the knowledge of a ‘just-deployment-ready’ CHW (assuming that the assessment is a good predictor of on-the-job performance). Evaluating on-the-job performance will also help PIH to establish the ‘learning curve’ i.e. the rate at which performance improves with experience.

A real test of the standard would require an evaluation of longer-term predictive validity, following-up all CHWs and independently rating their on-the-job performance. It would then be possible to determine whether the pass mark applied was a good predictor of safe practice (i.e. if all those passing were at least safe and vice versa). Clearly such a study is challenging to implement [23] and raises ethical issues, because it requires those who failed the assessment to be deployed to work with clients. However, the data required for such work could also be obtained from existing CHW program that have used post-training assessments without pass marks and which collect on-the-job performance data.

## Conclusions

As CHW program become more professionalized, ensuring that CHWs are at least safe to be deployed is an issue of increasing importance for CHW program providers. This requires the use of post-training assessments that are blueprinted to the program requirements, include well-written and quality-assessed items and where the borderline between pass and fail has been established using an appropriate method of standard setting. With this in mind, the study reported here outlines how program providers could start to implement standard setting for their assessments. We found that standard setting was a challenging process for panel members. Obtaining reliable standards requires that panel members gain experience of standard setting and we would therefore advocate the introduction of pilot standard setting exercises at an early stage in CHW program development. This would also have the advantage of increasing consistency amongst trainers and implementers as to the standards of knowledge and performance expected of CHWs and standard setting in practice can often help to identify problems with individual assessment items.

## Additional Files

The additional files for this article can be found as follows:

10.29024/aogh.2369.s1 Appendix 1. Post-training assessment with item-level standards.

10.29024/aogh.2369.s2 Supplementary data file. Standard setters’ ratings of importance and difficulty by item.

## Figures and Tables

**Table:** 

CHW	Community Health Worker
SEM	Standard error of measurement
