# Supplementary material for: Determining whether Community Health Workers are ‘Deployment Ready’ Using Standard Setting
Source: Ann Glob Health. 2018 Nov 5;84(4):630–9. doi: 10.29024/aogh.2369 (PMC6748218; doi:10.29024/aogh.2369)
Supplement: Appendix 1. — Post-training assessment with item-level standards. [file agh-84-4-2369-s1.pdf]

Appendix 1: CHW Household Model assessment (with answers and Ebel item standards – prior to adjustment using the Standard Error of Measurement)

Name: ________________________________ **Date:** ________________________

Catchment Area: ________________________

Village: ________________________________

**Male** or **Female**

1. **Which patients should be prioritized for monthly Home Visits?** (1 mark: standard 0.66)

   Patients who appear on the TRACE report.

2. **If a person tests positive for HIV, when should he/she start ARVs?** (1 mark: standard 0.75)

   Before the end of *2* weeks.

3. **List 3 symptoms of TB.** (3 marks: standard 2.07)

   1 mark for up to 3 of the following: Chest pain, cough for two weeks or more, fever, night sweats, lack of appetite, loss of weight, coughing up blood.

4. **When do CHWs stop visiting a TB patient daily?** (1 mark: standard 0.60)

   a. When she has completed 3 months of TB treatment.
   b. When she has completed 1 year of TB treatment.
   c. When she has completed her TB treatment.
   d. Never stop. CHWs visit TB patients daily, for life.

5. **If a sick patient is referred to the health facility, when should the CHW visit them to follow-up and check on the patient?** (1 mark: standard 0.69)

   Within *2 days* of the referral.

6. **List 3 of the non-communicable diseases (NCDs) included in the new Household Model Program.** (3 marks: standard 1.80)

   1 mark for up to 3 of the following: Hypertension, heart failure, asthma, diabetes, epilepsy, mental illness.

7. **What are the signs of malnutrtion that you would look for when screening a child under 6 months for nutritional status?** (3 marks: standard 1.59)

   1 mark for up to 3 of the following: can see outline of ribs. Loose skin on upper arms, loose skin on thighs, can see shoulder bones, flesh missing from buttocks.

8. **Until what age should you screen a child for nutritional status using the MUAC tape in the Household Model?** (1 mark: standard 0.60)

   Until they are *5* years old.

9. **If a woman of childbearing age is not on family planning, what should a CHW do?** **(1 mark: standard 0.63)**

    a. Give her a supply of contraceptive pills.
    b. Recommend the family planning method you think is most suitable for her.
    c. Refer her to the health provider for more information.
    d. Teach her about all the family planning methods.

10. **What should a CHW do following detection of a missed period?** **(1 mark: standard 0.53)**

    a. Re-visit the woman in her home to undertake a pregnancy test
    b. Refer the woman to the health facility for a pregnancy test
    c. Accompany the woman to the health facility for a pregnancy test.

11. **What should a CHW do when woman and baby are due for their 3rd day postnatal visit?** **(1 mark: standard 0.64)**

    a. Undertake the visit in the woman's home
    b. Refer the woman to the health facility
    c. Accompany the woman to the health facility.

12. **What material can you use to educate household members about STI symptoms?** **(1 mark: standard 0.64)**

    EITHER STI Counselling Card OR STI Job Card.

13. **When do Senior CHWs visit CHWs each month for the Supervision, Mentorship and Spot Check Visits?** **(1 mark: standard 0.57)**

    In the *1st* week of the month.

14. **Why would a CHW put an 'X' in the ANC column on the Household Register?** **(1 mark: standard 0.69)**

    a. The visit was cancelled.
    b. The woman attended that ANC visit.
    c. The woman does not have to go to that ANC visit.
    d. The woman missed that ANC visit.

15. **How should food supplements/Ready-to-use therapeutic food (RUTF) be used to treat a child with malnutrition?** **(1 mark: standard 0.59)**

    a. Eaten directly as it is only by the malnourished child.
    b. Eaten directly by all children in the household to prevent them from becoming malnourished.
    c. Mixed with porridge.
    d. Mixed with water and drunk.

16. **A child is confirmed as malnourished by the health facility. How is this indicated in the Household Register?** **(1 mark: standard 0.57)**

    By putting a tick in the 'malnutrition' column against the child's name (must give BOTH the correct column and note the need to specify which child is affected – no half marks).

17. **List 3 things that people can do to prevent hypertension.** **(3 marks: standard 1.54)**

    1 mark for up to 3 of the following: eat less salt, avoid alcohol, avoid/stop smoking, eat more vegetables, increase physical activity.

18. **Which 3 groups of people are considered most vulnerable to malaria?** **(3 marks: standard 2.01)**

    1 mark for up to 3 of the following: Children, pregnant women, HIV+ patients (PLWHIV).

19. **List 1 risk of a home birth you should educate a pregnant couple about.** **(1 mark: standard 0.62)**

    Any 1 of the following: HIV transmission between mother and child, both mother and child can die, mother can lose a lot of blood, both mother and child can get infection due to poor sanitation.

20. **Which of the following sections does NOT need to be completed on a Referral and Accompaniment form?** **(1 mark: standard 0.67)**

    a. Bottom half: patient details
    b. Bottom half: reason for referral and accompaniment
    c. Clinical section (section for the provider)
    d. Top half: reason for referral/accompaniment.
